# Industrial odour pollution and human health: a systematic review and meta-analysis

**DOI:** 10.1186/s12940-021-00774-3

**Published:** 2021-09-22

**Authors:** Victor Guadalupe-Fernandez, Manuela De Sario, Simona Vecchi, Lisa Bauleo, Paola Michelozzi, Marina Davoli, Carla Ancona

**Affiliations:** 1 Department of Epidemiology of the Lazio Regional Health Service, ASL Roma 1 (Italy), Via Cristoforo Colombo, 112, 00147 Rome, Italy; 2 Department of Preventive Medicine and Public Health, University of Valencia, 46010 Valencia, Spain

**Keywords:** Odour pollution, Residential exposure, Respiratory effects, Systematic review, Risk of bias

## Abstract

**Objective:**

To conduct a systematic review to evaluate the association between residential or occupational short- and long–term exposure to odour pollution from industrial sources and the health status of the exposed population.

**Methods:**

The searches were conducted in Medline, EMBASE and Scopus in April 2021. Exposure to an environmental odour from industrial sources in population resident near the source or in workers was considered. We considered outcomes for which there was a biological plausibility, such as wheezing and asthma, cough, headache, nausea and vomiting (primary outcomes). We also included stress-related symptoms and novel outcomes (e.g. mood states). Risk of bias was evaluated using the OHAT tool.

For primary outcomes, when at least 3 studies provided effect estimates by comparing exposed subjects versus not exposed, we pooled the study-specific estimates of odour-related effect using random effects models. Heterogeneity was evaluated with Higgins I^2^.

**Results:**

Thirty studies were eligible for this review, mainly cross-sectional (*n* = 23). Only one study involved school-age children and two studies involved workers. Only five studies reported odour effects on objective laboratory or clinical outcomes. Animal Feeding Operations and waste were the most common industrial sources.

The overall odds ratios in exposed versus not exposed population were 1.15 (95% CI 1.01 to 1.29) for headache (7 studies), 1.09 (95% CI 0.88 to 1.30) for nausea/vomiting (7 studies), and 1.27 (95% CI 1.10 to 1.44) for cough/phlegm (5 studies). Heterogeneity was a moderate concern. Overall, the body of evidence was affected by a definitely high risk of bias in exposure and outcome assessment since most studies used self-reported information.

**Conclusions:**

Findings underline the public health importance of odour pollution for population living nearby industrial odour sources. The limited evidence for most outcomes supports the need for high quality epidemiological studies on the association between odour pollution and its effects on human health.

**Supplementary Information:**

The online version contains supplementary material available at 10.1186/s12940-021-00774-3.

## Introduction

Odour emissions from industrial sites constitute a major health issue both for neighbouring residents and workers, mainly due to the olfactive nuisances generated during industrial production processes [1–3]. Nevertheless, little evidence is available on the impact of olfactory nuisance, compared to a large number of studies on the toxicity of the chemicals emitted by industrial plants such as wastewater treatment, livestock operations, composting facilities, landfills, paper and pulp mills or petrochemical industries. Odour pollution is regulated differently worldwide, and it is addressed at a national or municipal level by different policy frameworks [2, 4].

The olfactory function plays an important role in the detection of hazards in the environment, with the upper respiratory tract usually being the first point through which air pollutants enters the human body. Olfactory receptors of the nasal epithelium may detect odorant compounds inducing sensations in different ways. At elevated concentrations, odorant receptors may send signals via the olfactory and trigeminal nerve to the brain causing different reactions, also known as subjective symptoms. Odour sensations processed in the central nervous system may induce pleasant reactions, positive mood and emotions, but also negative responses including irritation, pain, sneezing, salivation, and vasodilation, ultimately resulting in nasal obstruction, bronchoconstriction, mucus secretion and inflammation. Malodours, mould or bad air quality have also been considered as environmental triggers of headaches, eyes irritation, and unusual tiredness [3, 5–10]. It is also important to note, that individuals’ sensory responses can vary due to physiological factors, age or sex, persistent exposure, perceived health risk, and various social factors [3, 5–11]. Odour-related symptoms seem more common in subjects with odour intolerance [5]. In fact, odour seems to not have an effect per se, but it is mediated by personal perception or annoyance [7, 10]. Annoyance is a psychological symptom that can be related to poor quality of life or negative mood states.

Several studies measure odour annoyance and monitor community impact by self-reporting of somatic symptoms, as well as objective health effects, commonly including respiratory inflammation and dysfunctions diagnosed by physicians. The population’s characteristics and health status have traditionally been considered in surveys and structured interviews when approaching odour assessment [12].

Estimations of odour frequency, intensity and hedonic tone in the environment differ substantially among countries, according to their odour regulations [1–4, 12] and there are no standardized methods for population and exposure assessment to be used for environmental epidemiology studies. Odours emissions are generally composed by complex mixtures of different volatile chemical compounds. Besides, the sensitivity of people and odours responses are different among individuals, hindering efforts to monitor and assess its health effects. In view of the above, it is considered that odour analytical tools are not sufficiently accurate [1, 5, 13–15]. However, there are some predictive and observational approaches that have been used to estimate population odour exposure, such as atmospheric dispersion models [2], distance to the source [12], frequency of odour events per year, sniff tests [1], chemical compounds analysis [16], population complaints monitoring (mean annoyance response or percent highly annoyed residents) [3, 10].

As a result, the overall impact on communities of odour emissions remains unclear and there has been a rising number of concerns and complaints regarding their possible health effects, ending up increasing the quantity of studies performed on this topic lately [4, 10].

We conducted a systematic review to evaluate the association between residential or occupational short- and long–term exposure to odour pollution from industrial sources and the health status of the exposed population.

## Methods/design

### Protocol and registration

Methods and inclusion criteria were registered for PROSPERO (registration number: CRD42018117449). The systematic review was reported according to the Preferred Reporting Items for Systematic Reviews and Meta-Analyses (PRISMA) statement guidelines [17].

### Eligibility criteria

Eligibility criteria were defined based on the PECO statement for the key elements (population, exposure, comparator and outcome). The population of interest were people of any age living near industrial sources or workers exposed to odour pollution in their workplace. We limited the definition of an industrial source as all areas hosting production and processing plants and facilities for chemicals, petrochemicals, manufacturing, waste or water disposal and/or treatment, cement, power generation, mining and metals, and we included other activities, such as production in industrial installations of pulp and paper, textile, slaughterhouses and livestock operations. We excluded studies which assessed the effects of exposure to indoor pollution sources. Studies were included if they captured exposure to an environmental odour from industrial sources including both objective and subjective measures. Nevertheless, we excluded studies that mainly were focused on malodorous toxic compounds emissions since it would be difficult to disentangle the odour effect from the toxic one. The comparison group represented any alternative to the exposed group; this was the minimum criterion for inclusion.

We considered primary outcomes for which there was a biological plausibility with the exposure, such as wheezing and asthma, cough, headache, nausea and vomiting. Odour annoyance has been considered both as a surrogate for exposure and outcome, having a strong association with odour intensity, hedonic tone and modelled odour from dispersion models [3]. We also considered secondary outcomes such as respiratory, stress-related symptoms and other stress-related consequences (e.g. cardiovascular sleep disorders), and also novel outcomes (e.g., mood) [5, 7, 18]. There was no prior restriction on the method used for outcome measurement. We excluded studies based on comparisons between odour exposure and odour discrimination and hedonic ratings. We included both observational and experimental study designs evaluating short- and long–term effects of odour pollution with an estimate health effect.

### Information sources and search

A preliminary search was conducted in bibliographic databases to identify subject terms and free terms relevant to the review question. Afterwards we developed a comprehensive systematic search strategy using a combination of Medical Subject Headings (MeSH) terms and free text terms. We revised the strategy appropriately for each database to take account of differences in controlled vocabulary and syntax rules. We implemented our search on April 2021, in Medline (via OVID, 1946 to search date) and EMBASE (1947 to search date). To identify additional studies, we screened the references list of the included studies and searched the related articles publication, through Scopus (2004 to search date). We set no date, and geographiclimits in our search strategy. We searched for grey literature by examining different university libraries, and national/government/NGO reports. Furthermore, we contacted experts seeking additional information about unpublished and published studies. The Ovid search string is presented in Additional file 1.

### Study selection

We uploaded search results into a reference management software (EndNote, Clarivate Analytics) to manage the screening and coding process. Two reviewers independently screened titles and abstracts of the records obtained from the searches (VFG, MDS). The full texts of potentially eligible studies were retrieved for evaluation and inclusion. Any discrepancy regarding inclusion or exclusion of a particular study between reviewers was resolved through discussion by a third reviewer (AC).

### Data collection process and data items

For studies that met inclusion criteria, two review authors independently extracted data using a data extraction form. Disagreements about the extracted information were resolved by discussion with the involvement of the research team when necessary. We contacted three authors for further information. All authors responded, one of them provided numerical data that had only been presented graphically in the published article, one provided a digital poster while the one remaining author could not provide the requested information.

Furthermore, the reviewers extracted data on study year and design from each study, sampling time frame, region or country where the study was performed, sample size (target, enrolled, follow-up rates) and characteristics of the population, description of the reference or control group, exposure definition (data sources) and assessment (e.g. distance from the facility, odour annoyance using a 5-point-likert scale or dispersion modelling odour assessment), health outcomes collected (methods used to measure the outcome), statistical approach performed by the authors to analyse the data, confounders or co-exposures (methods used to measure them and how they were considered in analysis), type of effect measure (Risk Ratio, RR; Prevalence Ratio, PR; Odds Ratio, OR; beta coefficients; absolute and relative change) and the 95% confidence interval (CI). When more than one effect measure was available from the same paper, the following sequential but alternative criteria (if the first does not apply, the second works and so on) were applied to choose the estimate to be extracted: that from the best adjusted model; the most significant one; the largest effect size. Information on funding and conflict of interest by the authors of the studies was extracted and considered when available.

### Risk of bias assessment in individual studies

The risk of bias (RoB) of included studies were independently assessed by two reviewers. Disagreements were discussed and resolved with a third author by consensus. We used the National Toxicology Program/Office of Health Assessment and Translation (NTP/OHAT) Risk of Bias Rating Tool for Human and Animal studies adapted to the review question (Program) [19, 20]. The tool considered nine domains: assessment of exposure, assessment of outcome, confounding (three elements), selection bias, performance bias, attrition/exclusion bias, outcome reporting bias and inappropriate statistical methods as an additional category for other potential threats to internal validity. Assessment of confounding was based on three elements: 1) the design or analysis accounting for confounding and modifying variables, 2) the adjustment for other concurrent exposures 3) the confounding variables measured reliably and consistently. The first two elements were evaluated according to the minimum set of confounders and co-occurring exposures considered a priori as relevant: sex, age, educational level/ socioeconomic status (SES)/ employment status, smoking status (active/passive) and any co-exposures (noise, traffic pollution, air pollution, indoor odour).

According to the OHAT risk-of-bias (RoB) tool, for each specific domain, a risk of bias “definitely low”, “probably low”, “probably high”, and “definitely high” was assigned and each paper was classified accordingly. We classify individual studies into an overall quality category, i.e. tiers from 1 (higher quality) to 3 (lower quality). The entire body of evidence was rated and grouped as having “not likely”, “serious” or “very serious” risk of bias, based on the RoB across studies and classification tiers. Confidence ratings were integrated on a standard evidence profile table.

### Data synthesis

Data patterns were explored and evaluated. Outcome-specific odour-related effects were extracted from each study into evidence tables. For primary outcomes, in cases in which at least 3 studies provided effect estimates by comparing exposed subjects versus not exposed, we pooled the study-specific estimates of odour-related effects. Effect estimates using different metrics (e.g. beta coefficients for unit increase in odour or risk ratio across multiple exposure categories) were not included in the meta-analysis. We pooled estimates using random effects models (Restricted Maximum Likelihood REML Method) [21]. Heterogeneity was evaluated with the I squared statistic [22], where 25%, 50% and 75% indicate a low, medium, high heterogeneity respectively. To assess if exposure assessment (subjective vs objective) was a potential explanatory factor for the heterogeneity, a stratified analysis was planned. We planned to assess the publication bias only for at least 10 effect estimates.

A narrative synthesis of the results was carried out for secondary outcomes.

Meta-analyses were carried out in STATA software version 14.0.

## Results

**Fig. 1:**
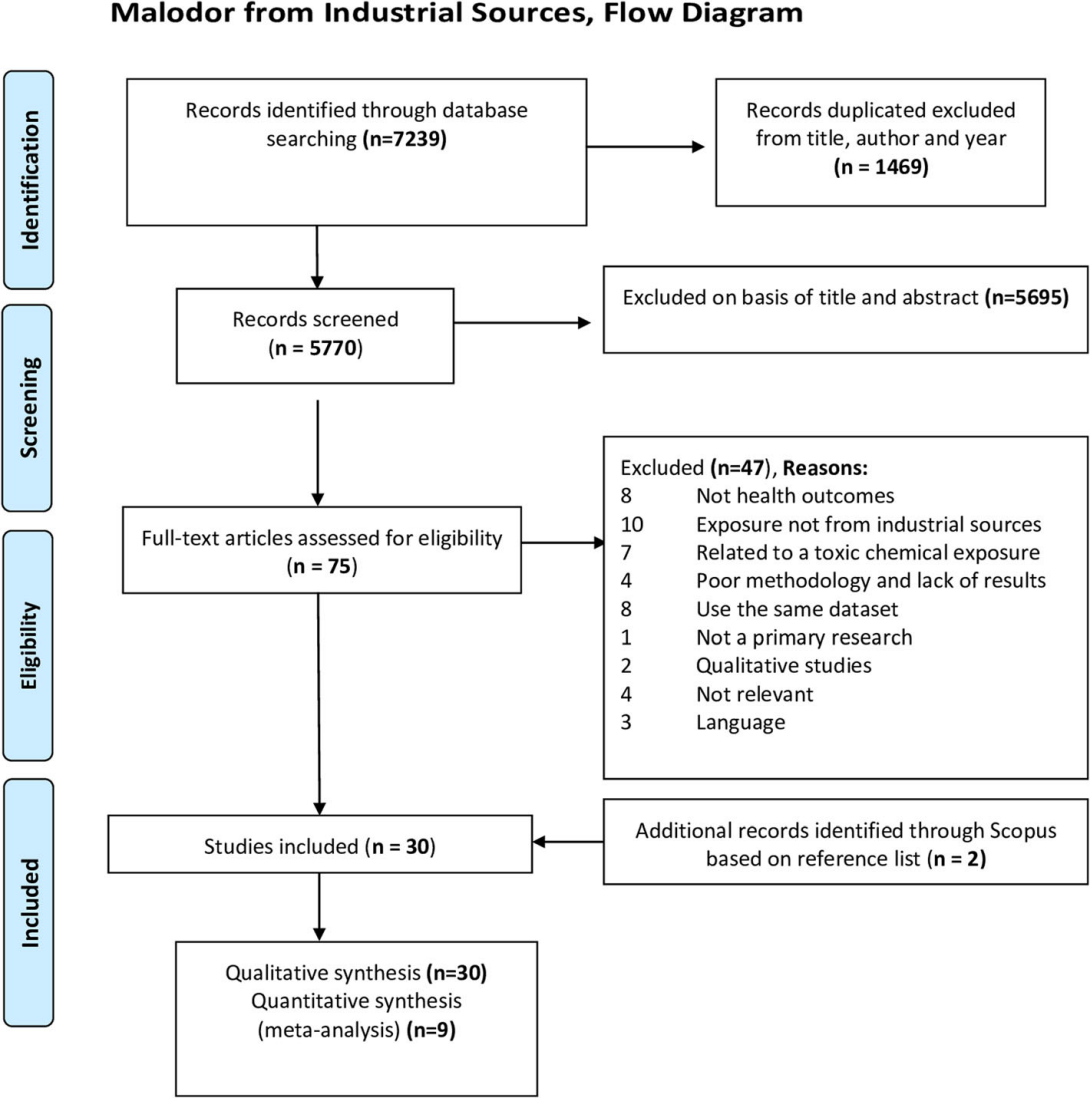
Systematic review on industrial odour effects on health - PRISMA flow diagram

Our search identified 5770 records after the removal of duplicates. Of these, 5695 were discarded on the basis of title and abstracts. No study was identified from grey literature sources. Seventy-five records were subsequently included in the full-text evaluation. From those, a total of 30 studies were included in the final synthesis with two additional records identified through reference list of the studies (Fig. 1).

**Fig. 2:**
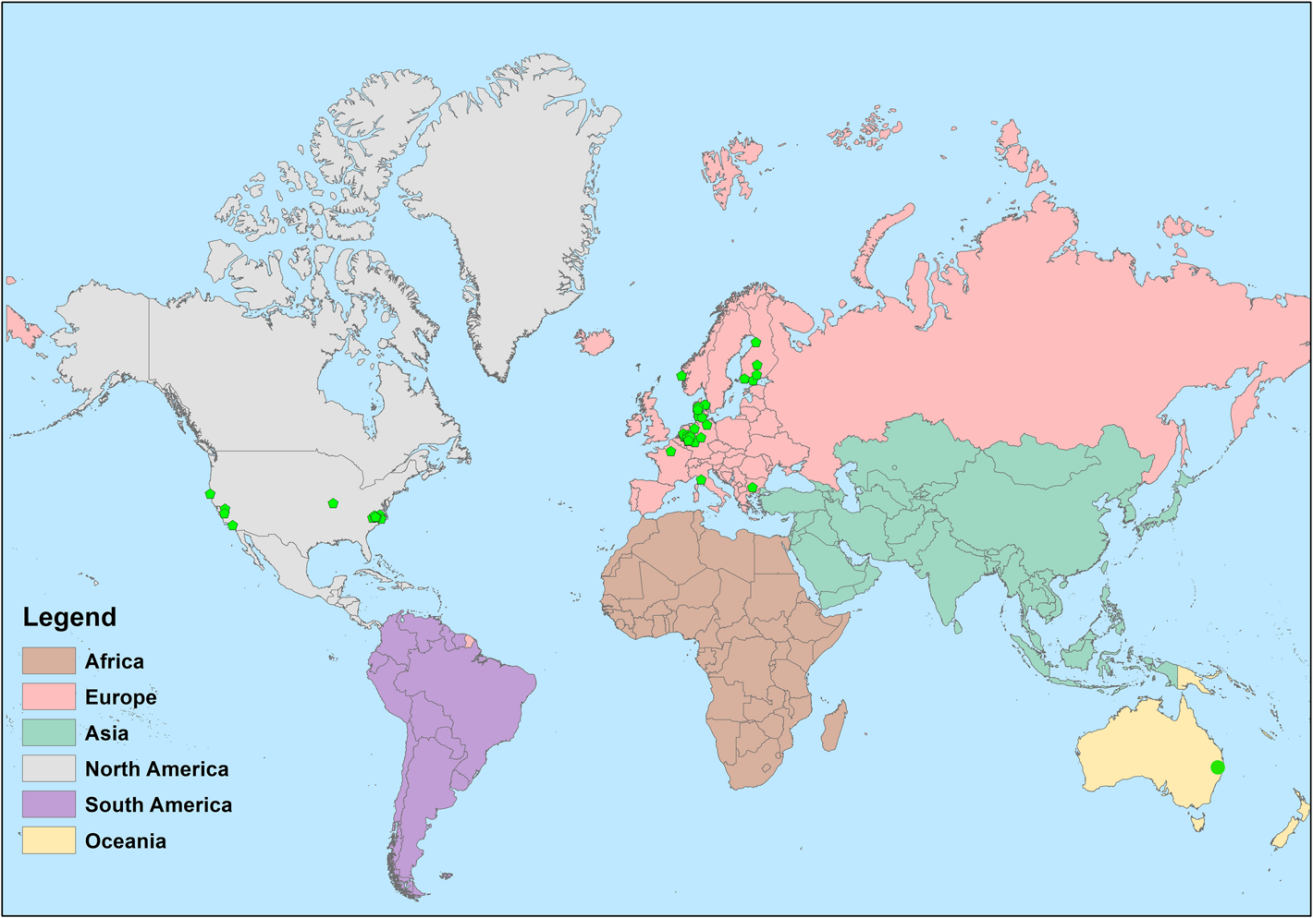
Geographic distribution of the included studies on industrial odour effects on health

Figure 2 shows the geographical distribution of the studies by country, with most sudies placed in Europe. Study size ranged from 15 to 58,169 subjects. The majority of the studies had a cross-sectional design (*n* = 23), while seven were panel studies [23–29].

The characteristics of the included studies, ordered by study design and by publication year (newer to older), are summarized in Table 1 and additional information are reported in Additional files 2 and 3.

**Table 1:** Summary of characteristics of studies included in the systematic review and meta-analysis by study design (cross-sectional, panel) and publication year (newer to older)

Study, Country, Study design	Industrial source	Study population, age group	Exposure assessment	Outcome assessment	Statistical analysis	Adjustment for confounders
Cross-sectional studies						
Kret 2018 [30], USA, Cross-sectional	Waste (landfill)	*N* = 343 adults households within a 3.2-km radius (173 exposed; 170 non-exposed)	Distance (km)	**Questionnaire**: self-reported prevalence of diseases and 12 months symptoms; odour annoyance (5-point Likert scale) **Groups:** Odour nuisances Lower respiratory symptoms Upper respiratory symptoms Gastrointestinal symptoms Mucus irritation General ill feeling	**Model:** n.a **Effect estimated:** n.a Weighted prevalence (95%CI)	Matching for percentage of white population and for 25+ population with education level at least high school. No effect estimate.
Hayes 2017 [31], Australia, Cross-sectional	Wastewater treatment Plant	*N* = 153 residents within a 3-km radius on two exposed (with a history of high or low number of complaints) and one control sites	**Questionnaire** (presence/absence of bad smells and odours impacting community)	**Questionnaire**: Self-reported psychological symptoms past week; odour annoyance (10-point scale) **Groups:** Mood states	**Model:** ANOVA **Effect estimated**: None	Social readjustment scale by Holmes and Rahe 1967 added as covariate
Tjalvin 2015 [32], Norway, Cross-sectional	Chemical Industry (Chemical explosion in an Industrial harbour)	*N* = 284 workers in 2008 and 203 in 2012 (exposed workers employed in 2008 and/or clean-up workers, proximity to the explosion ≤1 km; control workers) range of age 18–67	**Questionnaire**: Workers exposure history	Subjective Health Complaints (SHC) score **Groups**: General ill feeling Gastrointestinal symptoms Upper respiratory symptoms Immune function Cardiovascular problems	**Model**: Linear mixed effects models with random intercept and slope **Effect estimated**: Mean difference	Age, gender, smoking habits, educational level.
Tjalvin 2017 [33], Norway, cross-sectional (repeated survey of Tjalvin 2015)	Chemical Industry (Chemical explosion in an Industrial harbour)	*N* = 486 workers employed in 2008 (18% present during the explosion), in 2010 (*n* = 379), 2012 (*n* = 252) Adults aged 18–67 years	**Questionnaire**: Low/high odour score (% of months each participant noticed the odour in 2008)	**Questionnaire**: Subjective Health Complaints (SHC) score previous month; Impact of Event Scale-Revised (IES-R) previous 7 days Groups: General ill feeling Mood states	**Model**: Linear mixed effects models with random intercept and slope **Effect estimated**: Mean difference	Age, gender, smoking habits, educational level, absence/presence during the explosion (> 1 km or ≤ 1 km)
Boers 2016 [34], Netherlands, Cross-sectional	Animal feeding operations	*N* = 582 residents living near livestock farms Mean age = 51 years old (SD 13) (part of the population included in Hooiveld 2015)	**Calculated exposure**: 98th percentile of odour concentrations (OU_E_/m^3^) from Stacks dispersion model	**Questionnaire**: Self-reported odour annoyance (4-point scale) **Groups**: Odour nuisances	**Model**: multivariate logistic regression analysis **Effect estimated**: ORs(95%CI)	Age, educational level, indoor air pollution, asthma, or lower back pain
Hooiveld 2015 [35], Netherlands, Cross-sectional	Animal feeding operations	*N* = 753 adults, residents with asthma or lower back pain	**Questionnaire**: Self-reported odour annoyance (yes/no)	**Questionnaire**: Self-reported symptoms last month; general health (5-point Likert scale from bad to very good) **Groups**: Gastrointestinal symptoms General ill feelings Lower respiratory symptoms Upper respiratory symptoms Mood states	**Model**: Multiple ordinal logistic, logistic and Poisson regression analysis. **Effect estimated**: ORs(95%CI)	Smoking status, growing up at farm, age, gender, nationality, marital status, educational level, asthma or lower back pain, other environmental annoyances (noise, traffic and air pollution)
Baldacci 2015 [36], Italy, Cross-sectional	Waste (incinerator)	*N* = 1407 residents within 4-km radius from the incinerator and a control group. Mean age 44.4 (SD 22.1)	**Questionnaire**: Self-reported odour annoyance (no, slightly annoying, very annoying)	**Questionnaire**: Self-reported symptoms past 12 months. **Groups**: Lower respiratory symptoms Upper respiratory symptoms	**Model**: Multivariate logistic regression analysis **Effect estimated**: ORs(95%CI)	Gender, age, residence/incinerator distance, educational level, working position, smoking status, passive smoking, residential time, occupational exposure.
Blanes-Vidal 2015 [37], Denmark, Cross-sectional	Waste (biodegradable)	*N* = 454 Residents from six study areas in Denmark. Mean age 54 (SD 14)	NH_3_ concentration: log_e_ (NH^3^ exposure), NH_3_ exposure levels (< 2, 2–3, > 3 μg/m^3^), **Questionnaire**: Self-reported odour annoyance (no, slightly, moderately, very, extremely)	**Questionnaire**: Self-reported symptoms past 2 years, odour annoyance ((no, slightly, moderately, very, extremely) **Groups:** Odour nuisances Gastrointestinal symptoms General ill feeling Mood states	**Model:** multivariate logistic regression analysis. **Effect estimated:** ORs(95%CI)	Age, gender, smoking habit, job, time spent at home per week, existence of household residents below 18 years old, years living in the region, and acute and chronic respiratory conditions
Wing 2014 [38], USA, Cross-sectional	Sewage Sludge and Animal feeding operations	*N* = 158 adults, residents living near liquid TSS, 85 living near cake TSS, and 188 living in comparison areas	**Questionnaire**: Self-reported odour annoyance past six months (none/faint and moderate/strong/very strong)	**Questionnaire**: Self-reported symptoms past six months **Groups**: Gastrointestinal symptoms Mucus irritation General ill feeling Lower respiratory symptoms Upper respiratory symptoms Skin disorders	**Model**: Linear and poisson regression analysis **Effect estimated**: Mean factor score differences (95%CI) and PRs (95%CI)	Age, gender, race, educational level, smoking status, passive smoking, agricultural chemical odours and odours from burning
Aatamila 2011 [39], Finland, Cross-sectional	Waste (Landfills and composting sites)	*N* = 1142 residents within a 5-km radius of six different biowaste sites Range of age: 25–64 years	Distance zone (< 1.5, 1.5–3, > 3 km) **Questionnaire**: odour perception (4-point scale) stratified into sensitive vs not sensitive, odour annoyance (4-point scale) categorized as annoyed vs not annoyed	**Questionnaire**: Self-reported symptoms past 12 months **Groups**: Gastrointestinal symptoms Mucus irritation General ill feeling Lower and upper respiratory symptoms Skin disorders	**Model**: Logistic regression analysis **Effect estimated**: ORs(95%CI)	**Model 1**: adjusted for sex, age, educational level, Socio economic level and smoking **Model 2**: additionally, adjusted for odour sensitivity
Herr 2009 [40], Germany, Cross-sectional	Waste (composting sites)	*N* = 477 residents living “near” two composting sites. (263 EnvExp2 and 214 control group). Individuals aged ≥16 years old	Distance (km): EnvExp2 (odour-only exposed) and a control group	**Questionnaire**: Self-reported symptoms past 2 years **Groups**: Gastrointestinal symptoms General ill feeling Lower respiratory symptoms Mood states Skin disorders Cardiovascular symptoms	**Model**: Logistic regression analysis **Effect estimated**: ORs(95%CI)	Adjusted for age, gender, and educational level
Sucker 2008 [41], Germany, Cross-sectional	Industrial sites	*N* = 1434 adults from each household (the homemaking or the person spending most of the time at home)	**Questionnaire**: Log-values of odour frequency Intensity (6-point scale from “very slight” to “extremely strong”), Hedonic tone (9-point scale with values ranging from “-4” “extremely unpleasant” through “0” “neither pleasant nor unpleasant” to “+ 4” “extremely pleasant”)	**Questionnaire**: Odour annoyance; self-reported health complaints **Groups**: Odour nuisances General ill feeling Mucus irritation	**Model**: Logistic regression analysis. **Effect estimated**: ORs(95%CI)	Noise disturbance, length of residence, quality of residential area, tenant or owner, single/multiple houses, average time at home, perceived health, smoking habit, gender, age, marital status, educational level
Radon 2007 [42], Germany, Cross-sectional	Animal feeding operations	*N* = 5556 Residents from four rural town with high density of AFOs Mean age 33.6 (SD 7.4)	**Questionnaire**: Self-reported odour annoyance (4-point Likert scale from “not at all” to “strongly”)	**Questionnaire**: Self-reported symptoms during the week. **Clinical measurements**: Specific IgE to common allergens > 0.35 IU/mL, bronchial hyperresponsiveness to methacholine, forced expiratory volume in 1 s (FEV_1_) **Group**: Lower and upper respiratory symptoms Immune function and allergy	**Model**: Logistic and linear regression analysis **Effect estimated**: ORs(95%CI)	Age, sex, active and passive smoking, educational level, number of siblings and parental allergies. FEV_1_ additionally, adjusted for passive smoking during childhood
Mirabelli 2006 [43], USA, Cross-sectional	Animal feeding operations	*N* = 58,169 students of 265 schools within 3 miles of at least one AFO source Range of age: aged 12–14	**Questionnaire**: self-reported indoor and outdoor odours from schools (binary coded variable, “reported”/“no reported”)	**Questionnaire**: Current and past 12-month self-reported respiratory symptoms and medical care **Groups:** Lower respiratory symptoms General ill feeling	**Model:** Random-intercepts binary regression analysis **Effect estimated:** PRs (95%CI)	Age, race, socioeconomic status, smoking, school exposures and household exposures
Radon 2004 [44], Germany, Cross-sectional	Animal feeding operations	*N* = 2745 Residents living in rural towns close to intensive animal production Mean age 32.7 (SD 7.7)	**Questionnaire**: Self-reported odour annoyance (4-point Likert scale from “not at all” to “extremely”)	**QoL questionnaire**: Physical SF-12 score, emotional SF-12 score **Groups**: General ill feeling Mood states	**Model**: Multiple linear regression analysis **Effect estimated**: *β* (SE)	Age, gender, respiratory symptoms, smoking, living on or close to a farm and employment status.
Segala 2003 [45], Canada, Cross-sectional	Wastewater treatment plant	*N* = 2867 residents from 8 nearby towns. Distance zones: 3–4.5 km (*N* = 1003), mean age 47.5 (SD 15.2) 1.5–3 km (*N* = 1007), mean age 48.2 (SD 67.7) < 1.5 km (*N* = 857), mean age 49.8 (SD 15.1)	Distance zones (< 1.5, 1.5–3, 3–4.5 km) **Questionnaire**: Self-reported odour tolerance (“tolerant”, “moderately tolerant”, “intolerant”), odour annoyance (“annoyed with impact on health”, “annoyed without impact on health”, “not annoyed”)	**Questionnaire**: Self-reported symptoms past month and year **Groups:** Gastrointestinal symptoms Mucus irritation Lower respiratory symptoms Upper respiratory symptoms General ill feeling Cardiovascular problems	**Model:** Multivariate logistic regression analysis **Effect estimated:** ORs(95%CI)	Age, sex, educational level, active vs inactive, smoking status, family size, satisfaction with neighbourhood life
Georgieff 1999 [46], Bulgaria, Cross-sectional	Paper industry	*N* = 538 Residents from Stamboliisky town Range of age: 16–60 years old	**Questionnaire**: Self-reported unpleasant odour (yes/no)	**Questionnaire**: Self-reported symptoms **Groups:** General ill feeling Lower respiratory symptoms Mood states Immune function and allergy	**Model:** n.a. **Effect estimated**: n.a. Percentages (%) of number of reported somatic symptoms	None
Steinheider 1998 [47], Germany, Cross-sectional	***Nettetal study*** Fertilisers production plant ***Nörvenich study*** Pig rearing facilty	***Nettetal study*** (*N* = 250) ***Nörvenich study*** (*N* = 322) Adults aged ≥18 years old	***Nettetal study*** 1) Distance from the odour source Close: within 400–800 m Medium: 1600 m Far (control area): 6 and 3.5 km 2) 11-point graphic scale of Odour annoyance ***Nörvenich study*** 1) Log-values of odour frequency (odour hours/year). 34 observation points; 2) 11-point graphic scale of Odour annoyance	**Questionnaire**: Self-reported symptoms and odour annoyance (11-point graphic scale) Control variables (fever and asthma attacks) **Groups:** Odour nuisances Gastrointestinal symptoms General ill feeling Lower respiratory symptoms Mood states	***Nettetal study*** **Model:** Analysis of variance **Effect estimated:** None ***Nörvenich study*** **Model:** Linear regression analysis. **Effect estimated:** *β* (SE)	None
Steinheider 1993 [48], Germany, Cross-sectional	Industrial sites 1) Duisburg- chemical plant 2) Dortmund – iron/steel plant 3) Brühl – castiron factory and sugar refinery 4) Rodenkirchen – oil refineries	*N* = 1539 adults, living near of four cities in North Rhine-Westphalia. Duisburg (*N* = 400), *Dortmund* (N = 400), *Brühl* (*N* = 539), *Rodenkirchen* (*N* = 200)	Log-values of odour frequency (odour hours/year).	**Questionnaire**: Self-reported odour annoyance (11-point scale) **Groups:** Odour nuisances	**Model:** Multivariate linear regression analysis **Effect estimated:** n.a.	Age, sex, educational level, profession, length of residence and perceived health. Dortmund, Brühl and Rodenkirchen added coping strategies to the model
Lipscomb 1991 [49], USA, Cross-sectional	Waste (McColl waste disposal site)	*N* = 193 residents living nearby a disposal waste site Adults ≥22 years old	Exposure areas (high, medium, and low) based on an odour survey conducted in 1981	**Questionnaire**: Self-reported symptoms past 12 months **Groups**: Odour nuisances Gastrointestinal symptoms Mucus irritation General ill feeling Lower respiratory symptoms Upper respiratory symptoms Mood states Skin disorders Immune function and allergy	**Model**: n.a. **Effect estimated**: Crude PORs(95%CI)	None
Shusterman 1991 [9], USA, Cross-sectional	Waste	*N* = 2040 residents living near three hazardous waste sites in Southern California McColl. Acid petroleum sludge (*N* = 670) Operating Industries. Municipal and sewage (*N* = 514) Del Amo-Montrose. Residues from synthetic rubber manufacturing (*N* = 856)	Self-reported frequency of odour perception (“none”, “less than or equal to four times per month” and “greater than four times per month”	**Questionnaire**: Self-reported symptoms **Groups**: Odour nuisances Gastrointestinal symptoms Mucus irritation General ill feeling	**Model**: n.a. **Effect estimated**: PORs(95%CI)	None
Deane 1978 [50], USA, Cross-sectional	Refineries and other petrochemical industries	*N* = 291 Residents living in three residential areas nearby refineries and petrochemical plants	Exposure areas estimated by dynamic olfactometry: High (Area I), Moderate (Area II), Low (Area III).	**Questionnaire**: Self-reported symptoms **Groups:** Odour nuisances Gastrointestinal symptoms Mucus irritation General ill feeling Lower respiratory symptoms Upper respiratory symptoms Mood states	**Model:** n.a. **Effect estimated:** n.a. Frequency of self-reporting outcomes	None
Deane 1977 [51], USA, Cross-sectional	Paper industry	*N* = 140 Adults living in three residential areas nearby a pulp mill	**Exposure areas**: high (1–2 miles southeast of the mills), moderate (2–3 miles east of the mils), low (4 miles east of the mills)	**Questionnaire**: Self-reported symptoms **Groups**: Odour nuisances Gastrointestinal symptoms Mucus irritation General ill feeling Lower respiratory symptoms Upper respiratory symptoms Mood states	**Model**: n.a. **Effect estimated**: Frequency of self-reporting outcomes	Analysis were stratified by odour annoyance and gender
Panel studies						
Van Kersen 2020 [29], Netherlands, Panel (3 months)	Animal feeding operations	*N* = 82 adults COPD non smokers residents in the eastern part of the province of Noord-Brabant and the northern part of the province of Limburg (high prevalence of lifestocks)	NH_3_ concentration (μg/m^3^), **Questionnaire**: Self-reported odour annoyance (no, yes)	**Questionnaire**: Self-reported symptoms past 12-h **Clinical measurements**: Lung function (forced expiratory volume or FEV_1_ and peak expiratory flow rate or PEF) **Groups:** Lower respiratory symptoms Upper respiratory symptoms	**Model:** Generalized estimated equations (GEE) assuming a first order autoregressive (AR1) correlation structure **Effect estimated:** ORs (95%CI)	Adjustment for ambient temperature, relative humidity and day-in-study (linear trend), PM10; Restriction to non-smokers by study design
Wing 2013 [28], USA, Panel (2 weeks)	Animal feeding operations	*N* = 101 non-smoking residents living within 1.5 miles of an CAFOs source Adults aged ≥18 years old. Mean age 53.7 (19.2–89.5)	**Data-collection diary**: Self-reported odour annoyance (9-point Likert scale)	Systolic (SBP) and diastolic (DBP) blood pressure values **Groups**: Cardiovascular problems	**Model**: Linear fixed-effects models **Effect estimated**: *β* (SE)	Time-of-day (AM or PM)
Heaney 2011 [24], USA, Panel (14 days)	Waste (landfill)	*N* = 23 adults, residents within 0.75 miles of the landfill	**Questionnaire**: 12-h of self-reported odour annoyance (5-point Likert scale)	**Questionnaire**: Self-reported symptoms past 12-h **Groups**: Gastrointestinal symptoms Mucus irritation General ill feeling Lower respiratory symptoms Upper respiratory symptoms Mood states Skin disorders	**Model**: Conditional fixed effects logistic regression models **Effect estimated**: ORs(95%CI)	Time of day (AM/PM)
Schinasi 2011 [27], USA, Panel (14 days)	Animal feeding operations	*N* = 101 Non-smoking residents within 1.5 miles of an AFOs source Mean age 53.7 (19.2–89.5)	**Questionnaire**: 12-h of self-reported odour annoyance (9-point scale)	**Questionnaire**: Self-reported symptoms past 12-h **Clinical measurements**: Lung function (forced expiratory volume or FEV_1_and peak expiratory flow rate or PEF) **Groups:** Gastrointestinal symptoms Mucus irritation General ill feeling Lower respiratory symptoms Upper respiratory symptoms Skin disorders	**Model:** Conditional fixed effects logistic and linear regression analysis **Effect estimated:** *β* (SE)	Time of day (AM/PM)
Horton 2009 [25], USA, Panel (2 weeks)	Animal feeding operations	*N* = 101 Non-smoking residents within 1.5 miles of an AFOs source Mean age 53.7 (19.2–89.5)	12-h of self-reported odour annoyance (9-point scale)	**Questionnaire**: Self-reported information on mood states **Groups**: Mood states Odour nuisances	**Model**: Logistic mixed models with random intercepts. **Effect estimated**: *β* (SE) and ORs(95%CI)	Time of day (AM/PM)
Avery 2004 [23], USA, Panel (2 weeks)	Animal feeding operations	*N* = 15 residents within 2.4 km of an intensive hog operation facility Mean age 55.3 (SD 13.4).	**Questionnaire**: Self-reported odour annoyance (9-point scale, coded as a seven-level continuous variable)	**Clinical measurements**: Log salivary IgA concentration (*μ*g/ml) and secretion rate (*μ* g/ml) **Group**: Immune function and allergy	**Model**: Hierarchical mixed models **Effect estimated**: *β* (SE)	Day of data collection (1–14) and time of day (AM/PM)
Schiffman 1995 [26], USA, Panel (4 days)	Animal feeding operations	*N* = 88 Exposure group,: Mean age 52.0 ± 13.4 Control group: Mean age 51.7 ± 8.3	**Distance and duration**: Exposed living an average of 5.3 + 6.5 years near hog operations and comparison group	Profile of Mood States (POMS) factors and Total Mood Disturbance (TMD) score **Groups:** Mood states	**Model:** ANOVA **Effect estimated:** n.a.	adjusted by design (matching by gender, age, race, and education)

Only one study [43] involved a sample of school-age children (age range: 12–14 years). We observed a large heterogeneity in terms of type of industrial source, study population, measurements for exposure and outcome (i.e. objective or subjective) and type of outcomes. Regarding industrial source of exposure, 13 studies were conducted on Animal Feeding Operations (AFOs), 10 studies on waste (both solid and liquid waste), 2 were on multiple sites, and 6 were on other industrial exposure (e.g. paper, petrochemical plant).

Ten studies used distance from the source as proxy of odour exposure [26, 30, 31, 39, 40, 43, 45, 47, 50, 51].

Boers et al. 2016 estimated odour exposure using the Stacks dispersion model [34]. Lipscomb et al. [49] defined a measure of exposure based on odour zones adopted from an earlier survey. In addition, Blanes-Vidal [37] and van Kersen [29] included NH_3_ exposure as a proxy of odour exposure. In one study, two different exposure measures were used, distance and odour frequency measured by a group of trained panellists [47]. In 16 studies [9, 23–25, 27–29, 35, 37, 38, 41, 42, 44, 45, 47, 48], perceived level of exposure was rated labelling different scales (Likert-type scales and other alternatives) through questionnaires/interviews. Several studies used a dichotomous exposure of odour annoyance and/or odour perception, defined as presence/absence [9, 24, 29, 35, 36, 39, 41, 43, 45] .

Retrospective and self-reported information on outcomes, questionnaire-based, was the most widely used method for measuring primary outcomes. Most studies were related to both acute (e.g. symptoms, worsening of disease) and chronic outcomes (e.g. prevalence of diseases), with different timing of data collection, with past year prevalence in some studies [30, 36, 39, 43, 45, 49] or past 2 years [37, 40], or past 6 months prevalence [38], past 1 month [32, 33, 37], or current symptoms [9, 29, 31, 34, 42, 43, 45, 47].

On the contrary, the seven panel studies focused on short-term or acute outcomes, that varied on a daily base, such as symptoms of disease [24, 27], or mood [25, 26] or biological parameters such as lung/bronchial function [27, 29], immune function and allergy [23], blood pressure [28]. In addition, also a cross-sectional study [42] reported objective outcomes (bronchial hyperresponsiveness to methacholine, IgE concentration). In some studies information on timing of outcome data collection was not provided [9, 46–48, 50, 51].

Most cross-sectional studies took into account the potential confounding of age, sex, smoking status, educational level and/or SES [32, 33, 35–39, 41–45]. Panel studies [23–29] were adjusted only by time-varying variables (e.g. time of the day when outcome was measured) because they do not need to adjust for individual confounders since the study population serves as its own control. Eight studies [9, 26, 30, 46, 47, 49–51] did not account for any confounder and only one [30] reported to have matched exposed and control population by age, race and education level. One study on COPD patients restricted the study population to non-smokers [29].

**Fig. 3:**
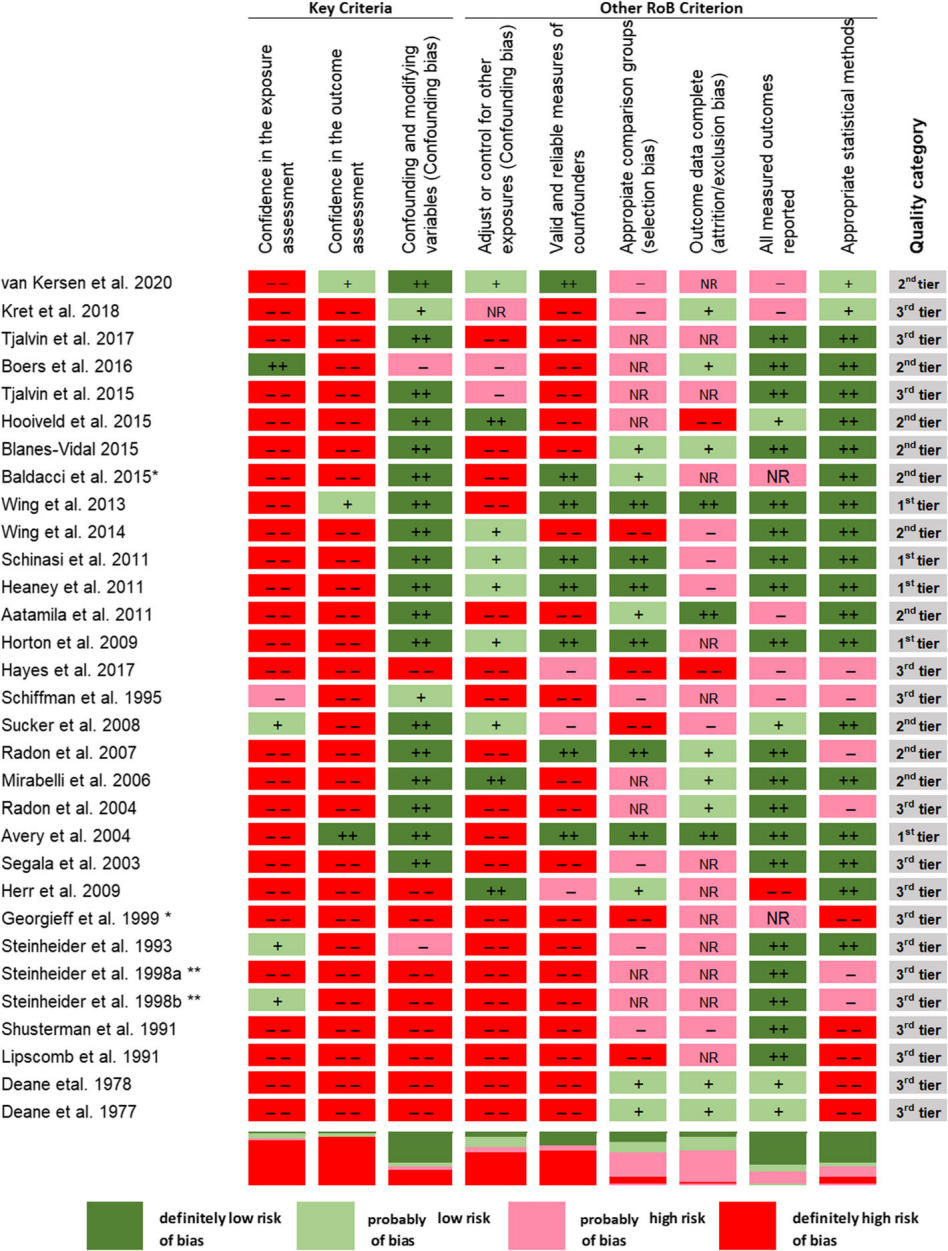
Studies on industrial odour effects on health according to the NTP/OHAT risk of bias approach. * conference proceeding; **Steinheider et al. 1998a Nörvenich study, Steinheider et al. 1998b Nettetal study

Figure 3 shows the results of evaluation of the risk of bias of the studies selected for the review. Overall, the body of evidence was affected by a definitely high risk of bias in exposure and outcome assessment since most studies used self-reported information. The study from Steinheider 1998 has been evaluated separately for the two study sites (Norvenich study labelled as a) and Nettetal study labelled as b)) [47]. Sixteen studies were classified in the worst quality level (3rd tier), 10 studies in the second (2nd tier) and five studies in the first category (1st tier).

Confidence in exposure and outcome assessment was very low in most studies. Only three studies were judged at low risk of bias since used objective outcome measures or only exposure from dispersion models [23, 28, 34].

As for confounding, adjustment with a minimum set of potential confounders was achieved in most studies for which the risk of bias was labelled low; 11 studies that did not account for any confounders were graded as “probably high” or “definitely high” risk of bias [9, 26, 30, 31, 34, 40, 46–51]. The second confounding element referred to the adjustment of other environmental exposure and in this case most studies did not adjust for concurrent exposures. For example, panel studies that only accounted for time of day (morning /evening) were considered as “probably high RoB” [23, 24, 28], due to the lack of adjustment for time-varying air pollution or noise. The third confounding element regarding validity and reliability of measures was characterized by a high risk of bias in most studies since information was mostly self-reported. Eight studies accounted for potential co-exposures, such as smoking, noise, indoor and/or outdoor pollution and were judged at very low/low risk of bias [24, 25, 27, 35, 38, 40, 41, 43].

The risk of selection bias resulted to be definitely high in five studies because the control group could not be defined as truly unexposed [31, 38, 41, 46, 49] or because personal attitude towards livestock farming could have influenced participation [29]. The risk of selection bias was probably high in most studies. Additionally, in six studies [32–35, 43, 44] and in the two Steinheider’s study sites [47] no information was provided as to whether selection of study participants resulted in appropriate comparison groups (Fig. 3). Regarding the attrition bias, in 10 studies [25, 26, 32, 33, 36, 40, 45, 46, 48, 49] and in the two Steinheider’s study sites [47] the information about loss of participants was unclear or incomplete, hence they were considered at “probably high” risk of attrition bias. Missing values related to outcome variables in the study were treated in the analysis. Only three studies were classified at “definitely low risk” of attrition bias [23, 28, 39]. Six studies were judged at probably or definitely high risk of reporting bias [26, 29–31, 39, 40], and, additionally, two studies [36, 46] were at unclear risk since outcomes were not reported with sufficient detail in the short communications. A probably low risk of reporting bias was found in Sucker et al. [41], after evaluating a previous publication of another part of the results [52].

Regarding the additional element of appropriate statistical methods, nine studies were judged at high risk (probably or definitely) since they provided only a descriptive analysis [9, 26, 31, 42, 44, 46, 49–51] and in the two Steinheider’s study sites [47].

Health outcomes were grouped as follows (Additional file 2): general ill feelings (e.g. headache, sleeping problems), gastrointestinal symptoms (e.g. nausea/vomiting, reflux), lower and upper respiratory symptoms (e.g. cough/phlegm, wheezing), immune function/allergy mucus irritation, skin disorders, mood states, cardiovascular problems, and odour nuisances (e.g. odour annoyance, risk perception). We ran meta-analyses for headache, nausea/vomiting and cough/phlegm. The Additional file 3 reported also the results not included in the meta-analyses of the association between residential or occupational, short- and long–term exposure to odour pollution from industrial sources and the risk. No measure of association was available for five studies [9, 30, 46, 50, 51] and for one of the locations (Nettetal) studied in Steinheider [47]. Only graphical results of Odds Ratios (and 95% confidence intervals) were provided for the association between NH_3_ exposure and prevalence of symptoms in the study of van Kersen 2020 [29].

Nineteen studies analysed general ill symptoms as health outcome of odour related effects [9, 24, 27, 30, 32, 33, 35, 37–41, 44–47, 49–51] (Additional file 3). All studies were on adults. Two studies were conducted among workers [32, 33].

**Fig. 4:**
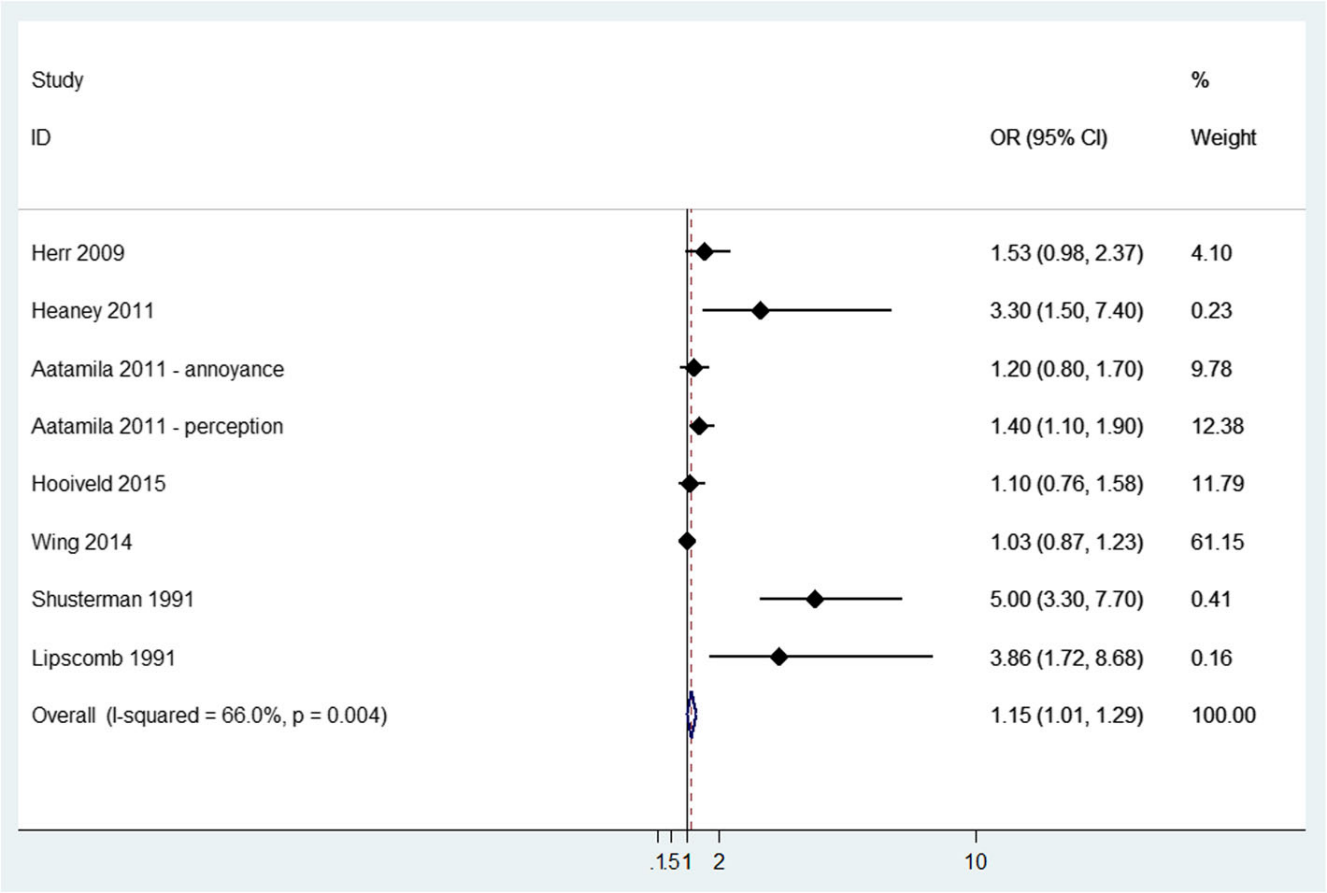
Forest plot of study-specific and pooled Odds Ratio (OR) and 95% Confidence Intervals (95%CI) of residential exposure to odour and headache in exposed versus non exposed subjects

Headache was the most common general ill symptom, being reported in 16 studies. Pooled analysis showed an increased risk of headache in exposed versus not exposed (OR = 1.15, 95% CI: 1.01 to 1.29) with moderate heterogeneity (I^2^ = 66%, *p*-value = 0.004) (Fig. 4). Among studies that were not included in the meta-analysis (Additional file 3), one study showed increasing headache prevalence [47] and two studies [37, 45] showed increasing risk in the highest exposure categories: at extremely annoyed compared to those who were not annoyed (OR = 3.65; 95% CI: 1.27 to 10.5); odour intolerant vs tolerant (OR = 2.64; 95% CI 2 to 3.5); group with complaints with impacts on health vs no complaint group (OR = 2.04; 95% CI 1.46 to 2.84).

Ten studies evaluated exposure to odour objectively [30, 32, 37, 39, 40, 45, 47, 49–51], reporting sparse evidence of association for dizziness [40], sleeping difficulties [47], fatigue [49], joint pain [39], fever past 12 months [39] and toothache [49].

Among studies evaluating exposure subjectively [9, 24, 27, 35, 37–39, 45, 51], most consistent associations were found for dizziness [24, 35, 37, 45], unnatural fatigue [37, 39, 45] and joint/muscular pain [39, 45].

Among exposed workers, significant higher total subjective health complaint (SHC) score [53] and the subjective neurological complaints score were found in exposed workers than in controls and these associations lasted for at least 3 years after the pollution was removed [32, 33].

Fifteen studies reported gastrointestinal symptoms [9, 24, 27, 30, 32, 35, 37–40, 45, 47, 49–51]. All studies were on adults. Only one study included workers [32].

**Fig. 5:**
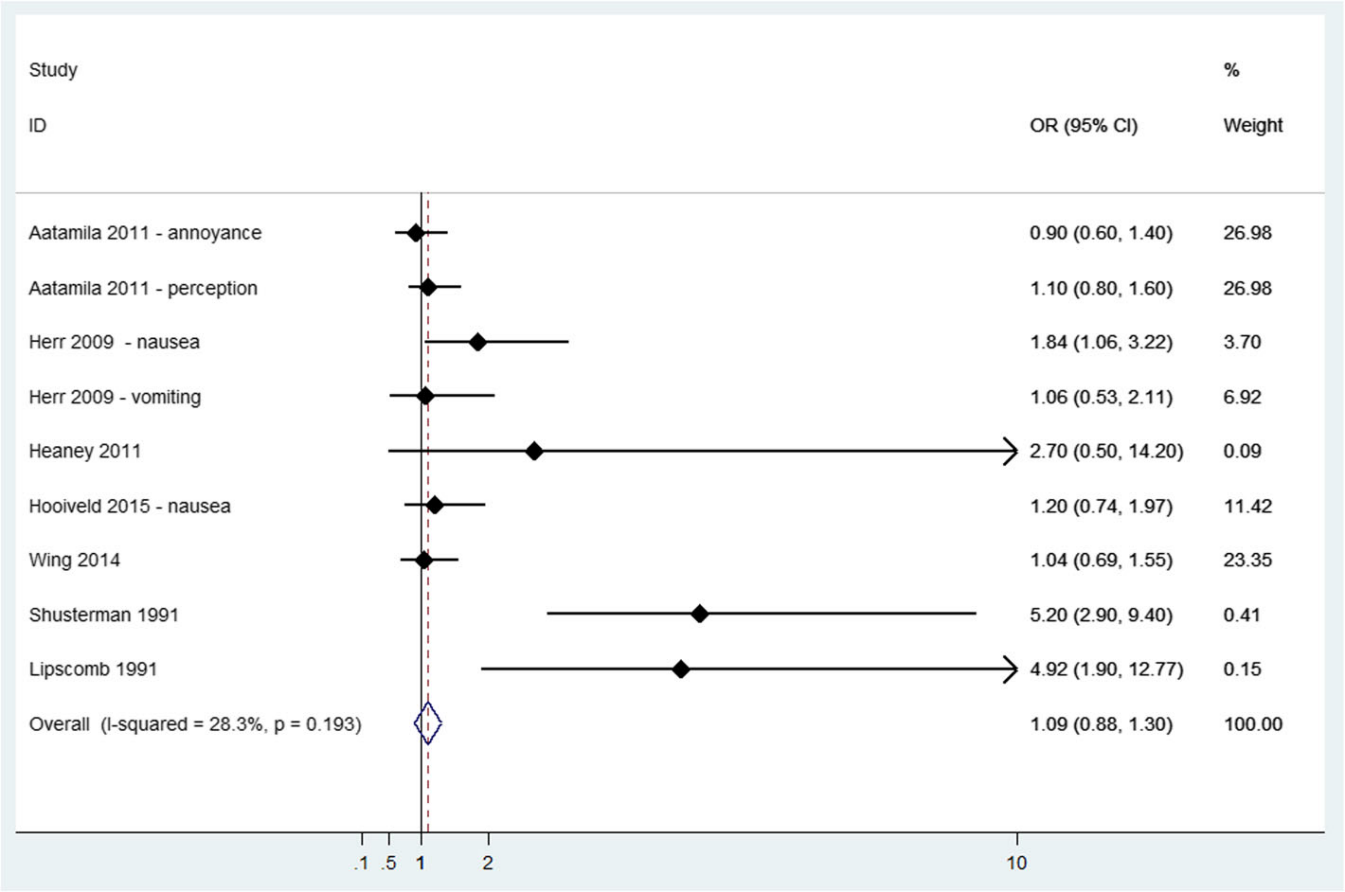
Forest plot of study-specific and pooled Odds Ratio (OR) and 95% Confidence Intervals (95%CI) of residential exposure to odour and nausea/vomiting in exposed versus non exposed subjects

The most frequent gastric symptom reported was nausea/vomiting. Seven studies [9, 24, 35, 38–40, 49] were feasible to meta-analysis (Fig. 5), showing an increased risk of these symptoms (OR = 1.09; 95% CI: 0.88 to 1.30) with a low heterogeneity (I^2^ = 28.3%; *p*-value = 0.193). Among studies not included in the meta-analysis (Additional file 3), self-reporting of vomiting, nausea or retching was significantly higher for increase in odour frequency in Nörvenich site [47] and in the study of Segala et al. [45] in the highest exposure categories: odour intolerant vs tolerant (OR = 3.52; 95% CI 2.14 to 5.8) and in group with complaints with impacts on health vs no complaint group (OR = 2.11; 95% CI 1.13 to 3.94). Estimates of the odour-nausea association tended to increase as the level of odour annoyance increased, but results were not significant in Blanes-Vidal et al. [37].

Among other gastric symptoms, eight studies measured exposure objectively [32, 39, 40, 45, 47, 49–51]. High exposure to odours was associated with greater prevalence of loss of appetite (OR = 4.27; 95% CI: 1.43 to 12.73) [49]. One study [47] showed a higher frequency of gastric symptoms (disgust, loss of appetite, stomach discomfort) when the frequency of odour exposure was increased. Another study [51] reported a significant trend by area among women who had reported frequently or occasionally constipation.

Seven studies evaluated exposure subjectively [24, 27, 35, 38, 39, 45, 51]. Segala et al. [45] reported more frequent diarrhoea in people with self-reported odour intolerance (OR = 2.18, 95% CI: 1.43 to 3.33) or experiencing malodour-related health complaints (OR = 2.83, 95% CI: 1.82 to 4.4); however, the same study did not report any significant association in people with complaints that were not related to health (OR = 1.08, 95% CI: 0.74–1.58). Aatamila et al. [39] found an increased risk of diarrhoea in the group with odour perception (OR = 1.3, 95% CI: 1 to 1.7) and with odour annoyance (OR = 1.2, 95% CI: 0.9 to 1.7). Statistically significant associations with stomach pain, gastrointestinal symptoms and constipation were reported in Hooiveld et al. [35].

There were no observed differences between groups for the gastrointestinal score among workers were observed [32].

Sixteen studies reported the association of lower respiratory symptoms with odour pollution [24, 27, 29, 30, 35, 36, 38–40, 42, 43, 45, 47, 49–51]. All studies were on adults except one [43]. No study was conducted on workers.

**Fig. 6:**
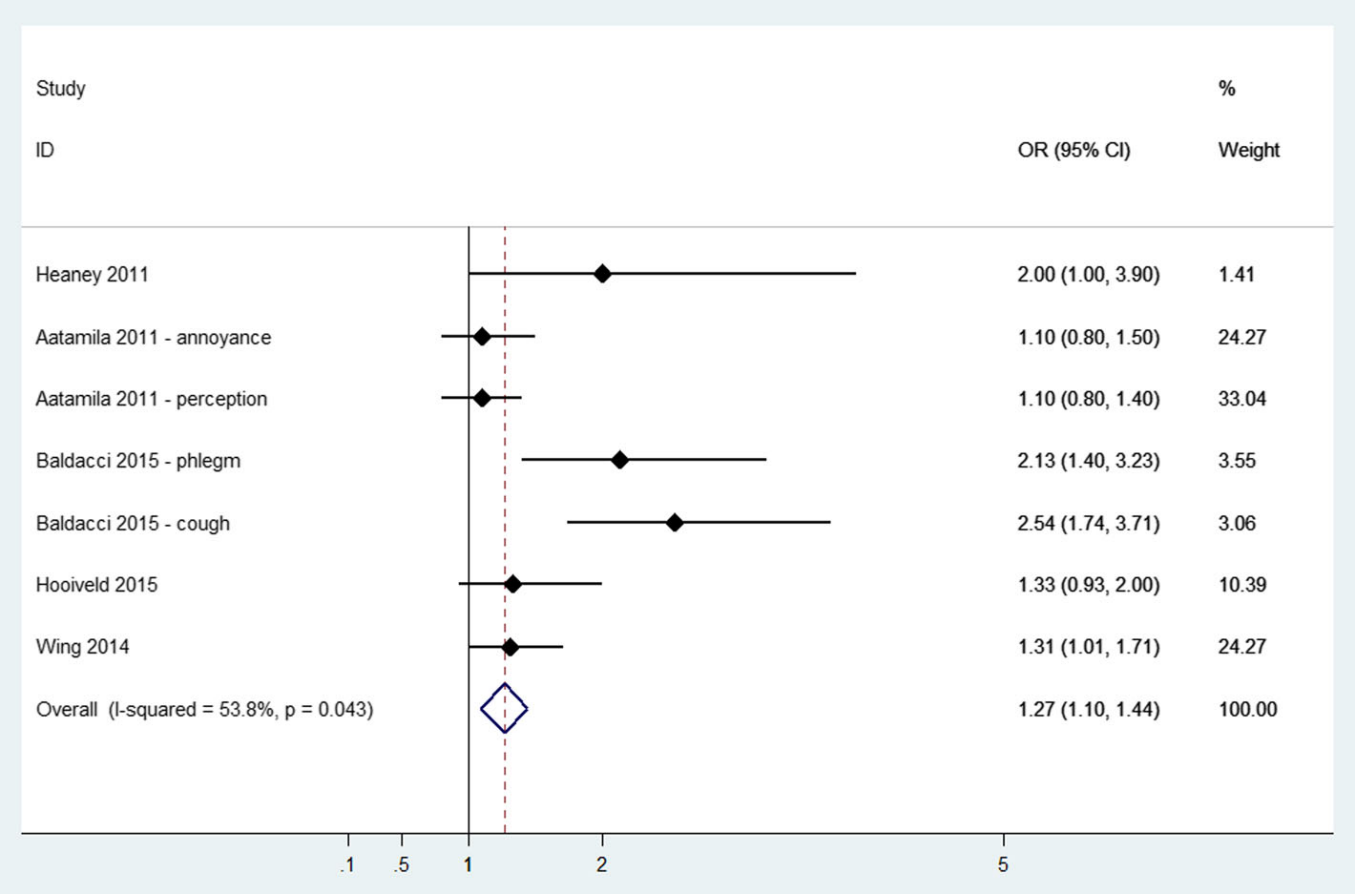
Forest plot of study-specific and pooled Odds Ratio (OR) and 95% Confidence Intervals (95%CI) of residential exposure to odour and lower respiratory symptoms

Eleven studies reported cough and phlegm as odour-related symptoms [24, 27, 30, 35, 36, 38, 39, 45, 47, 50, 51]. Pooled analysis showed an Odds Ratio of 1.27 (95% CI: 1.10 to 1.44) (see Fig. 6), with moderate heterogeneity (I^2^ = 53.8%, *p*-value = 0.043). Among studies that were not included in the meta-analysis (Additional file 3), self-reporting of cough/phlegm was significantly higher in the study of Segala et al. [45] in the highest exposure categories: odour intolerant vs tolerant (OR = 2.35; 95% CI 1.75 to 3.15) and in the group with complaints with impacts on health vs. no complaints (OR = 1.64; 95% CI 1.15 to 2.32) and in Aatamila et al. [39] the group of residents living closer to the waste site (distance< 1.5 km: OR = 1.3; 95% CI 1 to 1.8). Cough was significantly associated with odour frequency and even with odour annoyance after adjustment for odour frequency; however, no direct link was revealed between lower respiratory complaints and odour frequency after adjustment for odour annoyance [47]. Increasing reports of cough in the past 12 h related to 12-h mean odour were found in Schinasi et al. [27].

Among other respiratory symptoms, 10 studies reported exposure objectively [29, 30, 39, 40, 43, 45, 47, 49–51], mainly with distance as a proxy of exposure. Only three studies reported significant findings [43, 47, 49] for wheezing, asthma and shortness of breath. Mirabelli et al. [43] found school proximity within 3 miles of a swine CAFO was related to higher physician-diagnosed asthma (PR = 1.07; 95% CI: 1.01 to 1.14, mostly in non-allergic adolescents PR = 1.14; 95% CI: 1.01 to 1.26), asthma medication use (PR = 1.07; 95% CI: 1.00 to 1.15), asthma-related visit to a physician or an emergency department or hospitalization (PR = 1.06; 95% CI: 1.00 to 1.12), while for wheezing no clear association was found (Additional file 3) [43].

Between studies evaluating exposure subjectively [24, 27, 29, 35, 36, 38, 39, 42, 43, 45, 49, 51], eight reported significant health effects [27, 35, 36, 38, 39, 42, 43, 45]. Most consistent estimates were reported for asthma [36, 42, 45], while associations with wheezing were weaker. Wing et al. [38] showed odour from livestock facilities was significant related to difficulty breathing (PR = 1.52, 95% CI: 1.02 to 2.27) and increased the lower respiratory diseases score (mean difference = 0.28, 95% CI: 0.05 to 0.5) for moderate/strong/very strong odour group. According to Segala et al. [45], people complaining odour intolerance had a higher prevalence of self-reported respiratory infections (OR = 4.81, 95% CI: 3.24 to 7.14) or COPD (OR = 2.95, 95% CI: 1.84 to 4.73), and similar findings were found for the group with complaints with impacts on health vs. no complaints for COPD (OR = 2.05; 95% CI 1.21 to 3.49). People complaining about odours in terms of a health threat are found to be at a higher risk of enduring cough and COPD. Nonetheless, the precision of the effect estimate is lower in this sense. The included studies showed no association between odour and chest pain in the included studies.

Only three studies evaluated lung function and bronchial hyperresponsiveness [27, 29, 42]. A reduction in PEF and FEV1 with increasing odour was suggested in all studies [27, 42], however, 95% CIs included the null value, except than for the association between evening PEF (lag 0) and odour annoyance in the van Kersen study [29]. In addition, no associations were seen between self-reported odour annoyance and bronchial hyper-responsiveness to methacholine [42].

Eleven studies [24, 27, 29, 32, 35, 38, 39, 45, 49–51] presented data regarding associations between odours and upper respiratory symptoms (Additional file 3). All studies were on adults. Only one study was conducted on workers [32].

Regarding studies with objective exposure [29, 32, 39, 45, 49–51], no consistent associations were found between distance zones/exposure to NH_3_ and frequency of cold/flu, runny nose, nasal congestion and non-allergic rhinitis [29, 39, 45, 49–51].

Regarding studies with subjective exposure [24, 27, 35, 38, 39, 45, 51], a significant effect of odour with an increased risk for runny nose was found in only three [24, 27, 45]. In Segala et al. [45] the higher risk was found both in people with self-reported chemical intolerance (OR = 2.1, 95% CI: 1.59 to 2.78) and in people complaining of malodour in terms of a health threat (OR = 1.69, 95% CI: 1.22 to 2.32). A border line association was between cold/flu in last month and odour annoyance [35] (OR = 1.38, 95% CI: 0.97 to 1.99).

In the only study conducted on workers, there were no significant differences between the flu score in exposed subjects and the control group [32].

Five studies evaluated the effect of odour on the immune system and allergic sensitization by estimating IgE and IgA concentration and an allergy score obtained by questionnaires, using self-reported exposure [23, 42] or objective exposure [32, 46, 49], but no association with increasing odour exposure emerged.

Twelve studies evaluated odour effect on mucous membrane irritation [9, 24, 27, 30, 35, 38, 39, 41, 45, 49–51] (Additional file 3). Six studies were conducted on skin disorders [24, 27, 38–40, 49]. All studies were on adults. No study was conducted on workers.

The symptoms considered in the studies were: eye irritation, sore throat/burning throat, nose irritation, general irritation symptoms, skin irritation/itchy eczema.

Six studies evaluated the occurrence of irritation symptoms objectively by distance zones [30, 39, 40, 45, 49, 50]. Odour effects were found related to prevalence of dry throat within the last 12 months [39, 49], nose irritation [39], and skin irritation [49].

Regarding studies with subjective exposure [9, 24, 27, 35, 38, 39, 41, 45, 51], significant findings were found for eye irritation/burning eye [9, 24, 27, 39, 45] and for sore throat/dry throat/burning throat in five studies [9, 24, 27, 39, 45] (both odour tolerance and perception), for nose irritation/burning nose in two studies [24, 27], for nose/eye irritation symptoms in one study [41], and for skin irritation/rash in three studies [24, 27, 38].

Thirteen studies considered that malodour may have an impact on mood [24, 25, 31, 33, 35, 37, 40, 44, 46, 47, 49–51]. All studies were on adults. One study was on workers [33].

Six studies evaluated exposure objectively [37, 40, 46, 47, 49, 50]. Significant associations were only reported for nervousness, and difficulty concentrating [49].

Nine studies evaluated exposure subjectively [24, 25, 31, 33, 35, 37, 44, 47, 51]. Significant associations were found for all mood outcomes in Horton et al. [25], for nervousness, angriness, stress, unhappiness in Heaney et al. [24], and for sadness and stress-related symptoms in Hooiveld et al. [35]. In Blanes-Vidal et al. [37], a dose-response association between odour annoyance and difficulty concentration was found.

Considering the study on workers, participants in the high odour score group reported a higher post-traumatic score than those in the low odour score group, and these associations lasted for at least 3 years after the pollution was removed [33].

Three studies evaluated the effects of odour on cardiovascular symptoms and blood pressure [28, 40, 45]. Each unit of odour increase on an 8-point scale was associated with increases in diastolic blood pressure (mmHg) (OR = 1.26; 95%CI: 1.08 to 1.47), but not in systolic blood pressure [28]. No significant association was found in the other two studies [40, 45].

Ten papers [9, 25, 30, 34, 37, 41, 47, 49–51] investigated odour nuisances in the population regarding to their proximity to industries, odour perception, odour frequency or intensity, hedonic tone and NH_3_ exposure. All studies were on adults. No study was carried out on workers.

Regarding studies evaluating exposure objectively [30, 34, 37, 47, 49–51], odour annoyance showed a significant association with odour frequency [47], with NH_3_ concentration [37], as well as, with modelled odour exposure [34]. Moreover, three other studies showed a significant increase in odour nuisances in the closest areas to the odour source [30, 49, 50].

Regarding studies evaluating exposure subjectively [9, 25, 41], a significant dose–response association with odour annoyance was found in Sucker et al. [41], consistent across the different exposure measure (odour frequency, intensity, hedonic tone), aggravating the effect in subjects severely annoyed, and also in Horton et al. [25], the latter association was consistent across odour sources (livestock housings, slurry and manure, livestock farming in general).

## Discussion

This systematic review provides the state-of-art on the health effects of odour from industrial sources. Meta-analysis results showed that residential odour exposure was associated to an increased risk of headache and cough/phlegm, and to a borderline risk of nausea and vomiting. We found suggestive associations for the other outcomes investigated (e.g. asthma, mucus irritation, mood states) but evidence is sparse. Only two studies were carried out on occupational setting and they showed a statistically significant higher score of subjective complaints, neurological complaints and post-traumatic stress symptoms in exposed workers than in controls, and these associations persisted at least 3 years after the pollution was removed [32, 33].

The associations with headache, cough/phlegm and nausea/vomiting have a biological plausibility. Unpleasant odours are able to modulate autonomic system responses, such as vagal nerve inducing nausea or vomiting [5]. Another mechanism involves stress, consequent to environmental worry [18], and stress-related psychosomatic reactions such as chronic muscular tension, headaches, sleep disturbance. Chemicals responsible for odour may cause irritation, supporting the higher risk for cough/phlegm. Eye and nose irritation and asthma exacerbations can also be related to this odour-related irritation but only limited evidence was found in this review. Our review confirms the strong association between odour and annoyance confirming the potential mediation role on odour-related effects. We could not find any information on potential individual effect modifiers such as age, sex, educational level [54].

So far, only one other systematic review is available focused only on exposure from Animal Feeding Operation proximity providing little evidence of association between surrogate clinical outcomes and respiratory tract-related outcomes [55]. There is growing public attention on the topic at an international level as documented by the non-negligible number of studies retrieved in this review. Nowadays, there is also an effort by a variety of countries to classify odour as an atmospheric pollutant and regulate emissions by different policy frameworks worldwide [4].

Some limitations of our review should be mentioned. Formal test for publication bias was not carried out due to the limited number of studies included in the meta-analysis, but we cannot exclude this kind of bias and possibly other related biases (eg, language bias, citation bias, multiple publication bias) [56]. However, we expect that the comprehensive literature search, including grey literature, may have limited the impact of publications bias. The inclusion of small studies (less than 100 subjects) in our review suggests this bias is not a main concern. Meta-analytical estimates are affected by a moderate degree of heterogeneity due to difference among studies in terms of sources of exposure, population characteristics, study length. An additional concern derives from the multiple hypothesis testing that increases the probability of false positive results due to the multiplicity phenomenon as suggested also by other authors [55].

Moreover, the associations between odour and headache, nausea or cough need to be considered with caution due to the overall low quality of the studies for methodological problems of the observational study design.

Most of included studies had a cross-sectional design that can only provide a first hint of a hypothesized cause of a disease, but not a proof of causality [57]. Six studies used a panel approach, commonly used in air pollution epidemiology [58], representing one of the best options to study short-term health effects of odour although they can be affected by the drop-out bias and limited statistical power.

We used the approach proposed by the US National Toxicology Program [19, 20], one of the emerging approaches in the environmental (and occupational) health context, to evaluate the risk of bias of the body of evidence. Overall, 15 out of the 29 studies had a high risk of bias due to the limited confounding control, and exposure and outcome misclassification since most studies used self-reported information. On the contrary, five studies were at low risk of bias and the remaining nine showed an intermediate risk.

Regarding confounding, two aspects are worth of noting. In the present review, the most prevalent sources of odour were animal feeding operations and waste treatment sites. Therefore, exposure to air pollution from these industrial activities can be common and adjustment for concurrent environmental exposures is crucial to disentangle odour-related effects. Only few studies adjusted for these concurrent exposures such as noise, traffic, PM10, bioaerosol, pesticides [29, 35, 38, 41, 43], while another one stratified the study population to isolate the odour-only exposed group [40]. One of the included panel studies, without proper adjustment for concurrent environmental exposures, was downgraded to a high risk of bias [26]. Another issue emerging from the review is that in many included studies, confounders and co-occurring exposures were assessed by self-report.

Subjective exposure measures, such as odour rating and scores provided by participants, were used in most studies. Self-reported exposure is well known to be affected by information bias. The European Standard procedure for the measurement of odour concentration uses a dynamic olfactometry assessed by a panel [59]. However, none of the studies adopted this measurement method, but two studies followed other systematic standard methods for the assessment of odour frequency through panellist testing and olfactometers [41, 47]. However, it should be considered that the methods for assessing odour exposure should include also individual perceptions as effect modifier on odour impact on a population [12]. Odour perception, intolerance or annoyance or complaint [9, 35, 39, 40, 45] are adequate indicators to this aim. Some of the included studies have used distance as a proxy of odour exposure [30, 39, 40, 43, 45, 50, 51] and the Nettetal site studied in Steinheider et al. [47]. In our results, no consistent evidence of effects in the reporting of somatic symptoms was found by distance to the source of exposure. However, the bias should be non-differential across outcomes leading in some cases to underestimate true associations. Another exposure measure was the ammonia concentration in air [29, 37]. Although elevated levels of ammonia may cause irritative symptoms [60], the levels considered in the studies are several orders of magnitude lower than exposure limit in the workplace, 35 ppm for a short-term (15-min) exposure limit in the workplace, about 3000 times higher than the maximum level reported in Blanes-Vidal [37] and 2 hundred times higher than maximum reported in van Kersen et al. [29]. However, due to the complexity of odour mixtures, the use of ammonia as a surrogate for odour pollution, as clearly stated by the authors, represents a great limitation [37].

Included studies discussed the importance of using a standard objective method for exposure and outcome assessment in environmental epidemiology [12, 33, 34, 37, 41, 42], and some authors regarding other exposures mentioned dispersion modelling as a way out of this methodological issue [61]. Boers et al. was the only study that used air dispersion modelling as proxy of exposure [34]. Dispersion models include spatial characteristics (e.g. emissions, local meteorological conditions or topographical features, temperature, wind) which play a significant role in determining dispersion, concentration and intensity [61].

Only 5 out of 30 studies used objective outcome measurements such as lung/bronchial function [27, 29], immune function and allergy [23], blood pressure [28], bronchial hyperresponsiveness to methacholine [42]. Most studies lacked medical objective assessments and generally depended on participants recall of symptoms over different time periods that usually go from weeks to over the last 12 months. If in the same study, both outcome and exposure were self-reported, it may have occurred that exposed subjects, experiencing unpleasant odours, were also more worried about their health and therefore more prone to the reporting of health symptoms than non-exposed subjects, creating the case for differential misclassification of the outcome. Some studies tried to reduce this bias by not mentioning odour when presenting the survey or by the use of memory aids to help remember symptoms [38, 39]. In addition, respondents may be more likely to recall recent symptoms, also known as seasonal bias, having difficulty in remembering past events, related to the amount of time that has elapsed [30]. Response bias is a concern in most included surveys, both in terms of low participation rates and missing data to specific questions. That is why, future studies should attempt to address this issue by ensuring adequate response rates to the study or by controlling for non-response e.g. by weighting methods [62].

A recent European study collected all laws and regulations in efforts toward the management of odour impact in the communities, finding a heterogeneous picture (EU Project D-NOSES). Europe has included odours in the European Directive on industrial emissions (Directive 2010/75/EU) but at national level, laws and environmental guidelines are in place only in some countries such as Italy (Legislative Decree 152/2006). However, no specific public-health guidance is available. Wider considerations of odour exposure are expected to increase with increasing urbanization [12], e.g., due to waste disposal sites or intensive farming. It is clear that the effective prevention and response to protect public health is a matter of urgency. Addressing the odour problem is also an equity issue, since neighbouring residents of odour-polluted sites are most likely low-income groups, as it happens for air pollution [63].

## Conclusions

Findings from this systematic review underline the public health importance of odour pollution for population living nearby industrial odour sources. The limited evidence for most outcomes supports the need for high quality epidemiological research to better understand the association between odour pollution and its effects on human health. Exposure assessment is crucial and should be improved to overcome the lack of an objective and standardized method. Due to the strong mediation by odour annoyance and lack of evidence on individual effect modifiers, new studies should include also these aspects, for example studying vulnerable groups, such as children or pregnant women, and workers.

Considering the growing efforts in regulating odour pollution, it is important to define standardized methods to estimate its effects on population health, and to provide evidence-based guidance to bridge the gap also from a public health perspective.

## Supplementary Information

**Additional file 1.**

**Additional file 2.**

**Additional file 3.**

## Data Availability

The datasets used and/or analyzed during the current study are available as Additional file 3.
