# Supplementary material for: Industrial odour pollution and human health: a systematic review and meta-analysis
Source: Environ Health. 2021 Sep 22;20:108. doi: 10.1186/s12940-021-00774-3 (PMC8459501; doi:10.1186/s12940-021-00774-3)
Supplement: Supplementary file 1 — Additional file 1. [file 12940_2021_774_MOESM1_ESM.docx]

**Annex 1**

Search strategy (Ovid)

| **Population** | 1. (Work* or occupation*or residen* or living or population or populace or public or communit* or municipal* or neighbourhood* or neighbor* or neighbouring or urban or famil* or proximity or vicinity or location* or located or nearby or near or close or closely or surrounding or exposed).tw.) |
| --- | --- |
| **Exposure** | 2. ((((Odor* or malodor** or smell* or odour*) adj5 (waste* or incinerator* or production or landfill* or toxic* or emission* or pesticide* or fertilizer* or fume* or biowaste or composting or sewage or agricultur* or biomass* or environment* or farm*or feeding or treatment or rendering or livestock or animal or metal or industrial or petroleum or chemical or manufactur* or disposal or food or municipal or gaseous or organic or pollution)).tw) OR (((Odor* or malodor** or smell* or odour*) adj5 (operation*or factor* or refiner*or foundr* or facility* or plant* or industry* or processing or activity or activities or husbandry or surrounding or compound* or exposure or monitoring)).tw) OR (exp Odorants/)) |
| **Human studies only** | 1 AND 2 NOT  (exp animals/ not humans.sh.) |
